# Double-Detargeted Oncolytic Adenovirus Shows Replication Arrest in Liver Cells and Retains Neuroendocrine Cell Killing Ability

**DOI:** 10.1371/journal.pone.0008916

**Published:** 2010-01-27

**Authors:** Justyna Leja, Berith Nilsson, Di Yu, Elisabet Gustafson, Göran Åkerström, Kjell Öberg, Valeria Giandomenico, Magnus Essand

**Affiliations:** 1 Division of Clinical Immunology, Rudbeck Laboratory, Uppsala University, Uppsala, Sweden; 2 Department of Women's and Children's Health, University Hospital, Uppsala, Sweden; 3 Department of Surgical Sciences, University Hospital, Uppsala, Sweden; 4 Department of Medical Sciences, University Hospital, Uppsala, Sweden; Karolinska Institutet, Sweden

## Abstract

**Background:**

We have previously developed an oncolytic serotype 5 adenovirus (Ad5) with chromogranin-A (CgA) promoter-controlled E1A expression, Ad[CgA-E1A], with the intention to treat neuroendocrine tumors, including carcinoids. Since carcinoids tend to metastasize to the liver it is important to fully repress viral replication in hepatocytes to avoid adenovirus-related liver toxicity. Herein, we explore miRNA-based regulation of E1A expression as a complementary mechanism to promoter-based transcriptional control.

**Methodology/Principal Findings:**

Ad[CgA-E1A-miR122], where E1A expression is further controlled by six tandem repeats of the target sequence for the liver-specific miR122, was constructed and compared to Ad[CgA-E1A]. We observed E1A suppression and replication arrest of the miR122-detargeted adenovirus in normal hepatocytes, while the two viruses killed carcinoid cells to the same degree. Repeated intravenous injections of Ad[CgA-E1A] induced liver toxicity in mice while Ad[CgA-E1A-miR122] injections did not. Furthermore, a miR122-detargeted adenovirus with the wild-type E1A promoter showed reduced replication in hepatic cells compared to wild-type Ad5 but not to the same extent as the miR122-detargeted adenovirus with the neuroendocrine-selective CgA promoter.

**Conclusions/Significance:**

A combination of transcriptional (promoter) and post-transcriptional (miRNA target) regulation to control virus replication may allow for the use of higher doses of adenovirus for efficient tumors treatment without liver toxicity.

## Introduction

Virotherapy is an emerging approach to treat cancer. It utilizes genetically engineered viruses for selective infection and killing of tumor cells while leaving normal cells relatively unharmed. Today, transcriptional and transductional targeting are the two main strategies to selectively restrict adenovirus activity to tumor cells. Transcriptional targeting is mainly achieved by replacing an endogenous viral promoter sequence, e.g., the adenovirus E1A promoter, with a mammalian tumor- or tissue-specific promoter [1], [2]. This strategy restricts virus replication to target cells where the promoter is active. Transductional targeting concerns genetic or chemical alteration of capsid proteins for selective infection of tumor cells [1], [2]. Recently, a novel strategy based on the gene silencing mechanisms utilized by endogenous microRNAs (miRNAs) has been exploited to control viral replication. miRNAs are small, noncoding RNA molecules 20–24 bp in length that bind to mRNA in a sequence-specific manner. Partial complementarity in base-pairing between miRNA and target mRNA can act to suppress mRNA translation, but upon high sequence homology, miRNA cause catalytic degradation of target mRNA [3], [4], [5]. Naldini and colleagues were the first to use miRNA target (miRT) sequences to specifically suppress transgene expression from lentiviral vectors in hematopoetic cells or hepatocytes [6], [7]. Kelly et al were able to restrict replication of an oncolytic coxsackievirus (CVA21) by incorporation of miRT sequences recognized by a muscle-specific miRNA. This reduced replication of the CVA21 virus in normal muscle tissue and resulted in reduced muscle toxicity without compromising the tumor cell-killing ability [8]. Two recent publications have described miR122-detargeting of the human serotype 5 adenovirus (Ad5) to reduce adenovirus-induced liver toxicity [9], [10]. Both publications use the wildtype E1A promoter to control E1A and demonstrated that incorporation of miR122 target sequences in the 3′UTR of E1A gene reduces E1A expression in hepatic cells. Quantification of adenoviral replication was not examined in these papers.

We have previously described an oncolytic Ad5 virus where the chromogranin-A (CgA) promoter controls expression of the adenoviral E1A gene, Ad[CgA-E1A] [11]. CgA is a protein in secretory granules of neuroendoocrine cells that serves as a precursor of several biologically active peptides. The CgA gene is highly expressed in neuroendocrine tumors and CgA is known to be a sensitive and specific tumor maker for neuroendocrine tumors [12], [13], [14]. We demonstrated that the CgA promoter is functional when inserted in an adenovirus genome and retains selectivity for neuroendocrine cells, including neuroblastomas and neuroendocrine tumors of the ileum, also known as midgut carcinoids [11]. However, despite the fact that CgA is not expressed in the normal liver cells, Ad[CgA-E1A] shows weak activity in freshly isolated hepatocytes [11]. Since liver toxicity is potentially the most serious adverse event of adenovirus-based therapy, we have been investigating means to reduce Ad[CgA-E1A] activity in hepatocytes. In this study, we present further modification of Ad[CgA-E1A], by introducing miRT sequences for the liver-specific miR122 in the 3′UTR of E1A to down-regulate E1A expression and thereby viral replication in hepatocytes. To our knowledge this is the first study to demonstrate that a combination of transcriptional (promoter) and post-transcriptional (miRNA target) regulation to control adenovirus replication leads to tighter control than either of the two regulatory mechanisms alone.

## Results

### Liver-Specific Expression of miR122

miR122 is specifically expressed in hepatocytes and it is the most abundant miRNA molecule expressed in the adult liver where it makes up 70% of all miRNAs [15]. High and specific miR122 expression was confirmed in normal human liver, Balb/c mice liver and in the hepatoma cell line HuH7.5 (Supporting Figure S1). The miR122 expression level was approximately 7-fold higher in human hepatocytes than in HuH7.5, while miR122 was not expressed in the hepatoma cell line HepG2 or in the non-hepatic neuroendocrine tumor cells lines BON (pancreatic carcinoid), SH-SY-5Y, SK-N-BE(2), Kelly (all neuroblastomas) or in primary carcinoid cells (Supporting Figure S1). miRNA studies have shown that the strand used for recognition of target mRNA is the one, whose 5′ end is less tightly paired [16]. Based on our results and those studies, we designed a target sequence with six tandem repeats with perfect complementarity to the mature strand of the miR122 duplex.

### A Recombinant Adenoviral Vector with miR122 Target Sequences Shows Markedly Reduced Activity in Human Hepatocytes In Vitro and Mouse Liver In Vivo

**Figure 1:**
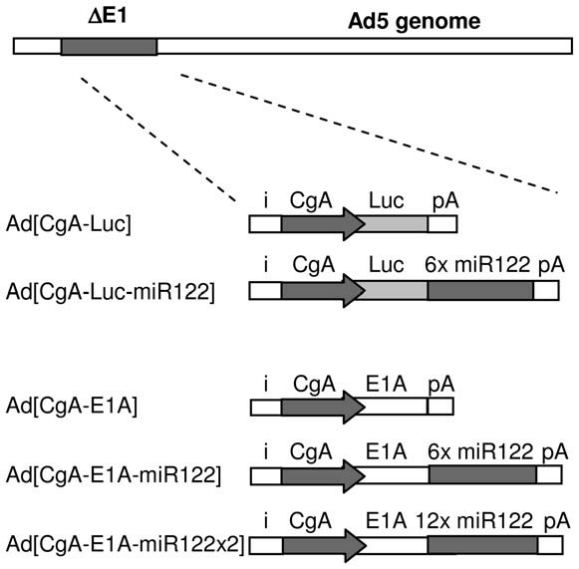
Schematic illustration of recombinant adenoviruses. Ad[CgA-Luc] and Ad[CgA-Luc-miR122] are E1-deleted Ad5-based vectors with luciferase gene expression controlled by the human CgA promoter. Ad[CgA-Luc-miR122] has six repeats of the miR122 target sequence in the 3′UTR of the luciferase gene. The oncolytic adenovirus Ad[CgA-E1A], which has been previously described, has E1A expression under control of the CgA promoter. Six and twelve repeats of the miR122 target sequence were inserted in the 3′UTR of the E1A gene in Ad[CgA-E1A-miR122] and Ad[CgA-E1A-miR122x2], respectively.

**Figure 2:**
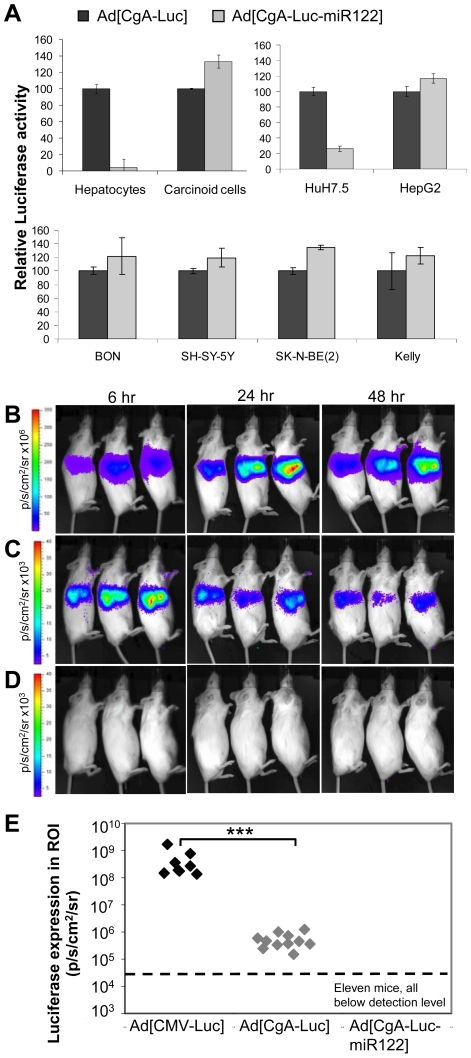
Specific silencing of luciferase expression in liver cells by miR122 *in vitro* and *in vivo*. (A) Freshly isolated primary cells and cell lines were transduced with Ad[CgA-Luc] and Ad[CgA-Luc-miR122] at MOI 10 and plated in 12-well plates. Cells were harvested after 48 hours and luciferase activity and protein concentration were determined. Luciferase activity was calculated as RLU/mg and expressed in relation to Ad[CgA-Luc], which was set to 100% for each cell type. Data are presented as the mean values of triplicates ± std dev. (B-E) Balb/c mice were injected i.v. with 5×10^10^vp of Ad[CMV-Luc] (B), Ad[CgA-Luc] (C) or Ad[CgA-Luc-miR122] (D). Luciferase activity was monitored with the IVIS-100 system 6, 24 and 48 hours after injection. (E) Comparison of luciferase activity in mice liver after i.v. injection of Ad[CMV-Luc], Ad[CgA-Luc] or Ad[CgA-Luc-miR122] (*** p<0.0001).

We constructed two serotype 5 adenoviral vectors with CgA promoter-controlled luciferase expression with or without miR122 target sequences in the 3′UTR of luciferase as presented in Figure 1. Transduction of freshly isolated hepatocytes with Ad[CgA-Luc-miR122] demonstrated more than 99% reduction of luciferase activity, compared to Ad[CgA-Luc] while no reduction was observed in freshly isolated carcinoid cells (Figure 2A). Luciferase expression was reduced by approximately 80% in the miR122-positive hepatoma cell line HuH7.5, while no reduction was observed in the miR122-negative hepatoma cell line HepG2 (Figure 2A), which strongly indicates miR122-specific silencing. The raw data on luciferase expression levels in the primary cells and cell lines are presented in Supporting Figure S2. Transfection with siRNA that mimics miR122 reduced luciferase expression in HepG2 to below 10% while a negative control siRNA only reduced the expression to 75%, giving further evidence of miR122-specific silencing (Supporting Figure S3). Suppression of luciferase activity was as expected not observed in the neuroendocrine tumor cell lines BON, SH-SY-5Y, SK-N-BE(2), Kelly (Figure 2A) while addition of siRNA mimicking miR122 silenced transgene expression (Supporting Figure S3).

The potency of miR122-silencing was next examined *in vivo*. miRNA are know to be evolutionary conserved across species and *H. sapiens* and *M. musculus* miR122 precursors are clustering in the same clade, which implicates recognition of similar target sequences. Mice were injected intravenously (tail vein) with a single dose of 5×10^10^ vp of the following vectors: Ad[CMV-Luc], Ad[CgA-Luc] and Ad[CgA-Luc-miR122]. Six, 24 and 48 hours after injection, luciferase activity was measured using the IVIS-100 system. In agreement with numerous other studies in mice, we found that the liver is the primary target of Ad5-based vectors after intravenous injection. Luciferase activity was observed in all mice injected with Ad[CMV-Luc] and Ad[CgA-Luc] (Figure 2B and C) already after 6 hours, while no luciferase activity was detected in any of the mice injected with Ad[CgA-Luc-miR122], despite long (10 minutes) exposure times (Figure 2D). Analysis after 6, 24 and 48 hours showed that the expression pattern does not change over time (Figure 2B, C and D). The viral vector with the constitutively active CMV promoter, Ad[CMV-Luc], resulted in significantly higher luciferase expression (3-logs) in the mouse liver compare to Ad[CgA-Luc] (Figure 2E).

### Reduced E1A Protein Expression and Arrest of Viral Replication Occurs Only in miR122-Expressing Hepatic Cells

**Figure 3:**
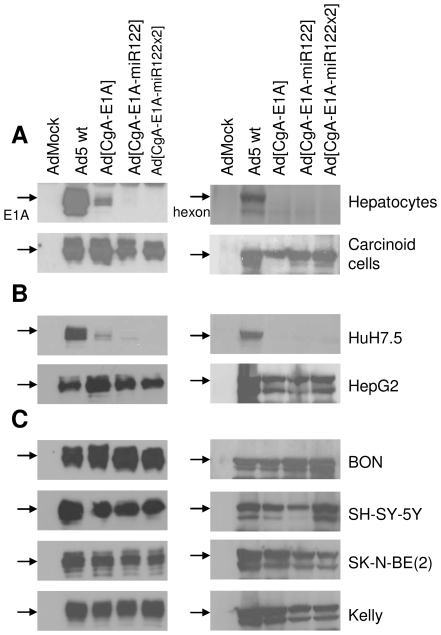
miR122-mediated suppression of adenoviral protein expression in hepatic cells. Primary cells (A), hepatoma cell lines (B) and neuroendocrine tumor cell lines (C) were transduced with viruses at MOI 1 (neuroblastoma cell lines at MOI 10). Total protein lysates were prepared after 48 hours and 50 μg of samples were resolved by SDS-PAGE. E1A was detected by Western blotting using an anti-E1A antibody. The polyclonal anti-Ad serum (α-Prage) was used to detect adenoviral capsid proteins. Hexon protein expression is shown to visualize production of progeny virions.

We next constructed two miR122-targeted oncolytic adenoviruses, Ad[CgA-E1A-miR122] and Ad[CgA-E1A-miR122×2], where E1A gene expression is driven by the CgA promoter and further controlled by six or twelve tandem repeats of miR122 target sequence in the 3′UTR of E1A (Figure 1). Transduction of normal hepatocytes with Ad[CgA-E1A] resulted in far lower E1A protein expression than wild-type Ad5 (Ad5 wt) and E1A expression was completely suppressed after transduction with Ad[CgA-E1A-miR122] and Ad[CgA-E1A-miR122×2] at MOI 1 (Figure 3A), indicating very efficient silencing. In comparison, transduction of carcinoid cells yielded strong E1A protein expression for all viruses (Figure 3A). When miR122-positive HuH7.5 was transduced with Ad[CgA-E1A] weak E1A expression was observed while E1A expression was completely abolished after transduction with the miR122-detargeted viruses (Figure 3B). E1A protein expression was not suppressed in miR122-negative HepG2 (Figure 3B). Moreover, in hepatocytes and HuH7.5, expression of hexon protein was strongly reduced (Figure 3A, B), which indicates that reduced E1A expression leads to production arrest of progeny virions. The CgA promoter strongly supports E1A protein expression, with efficiency comparable to the wild-type E1A promoter of Ad5 wt, in neuroendocrine tumor cells and expression was not altered by incorporation of miR122 target sequences in the viral genome (Figure 3C).

**Figure 4:**
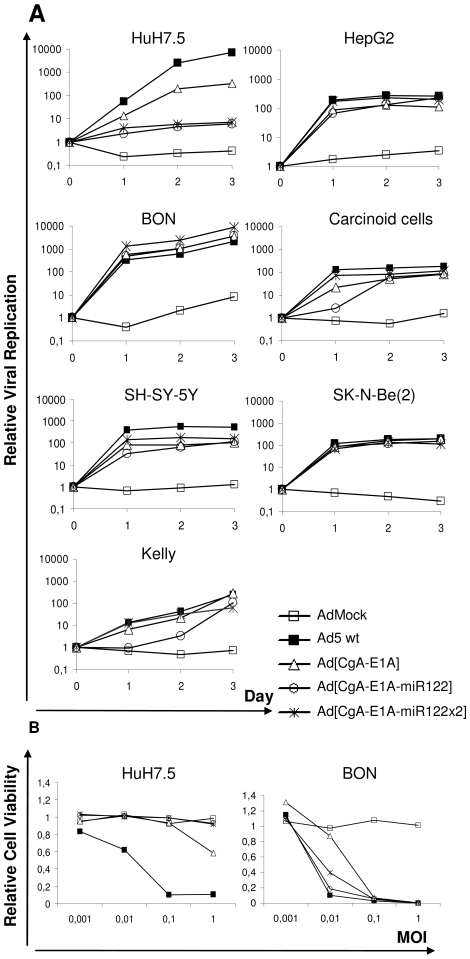
Replication arrest and reduced cytolytic activity in hepatic cells for adenoviruses carrying miR122 target sites. (A) Cells were transduced with viruses at MOI 1 (neuroblastoma cell lines at MOI 10). Viral DNA was isolated 2 hours, 1, 2 and 3 days after transduction. QRT-PCR was performed to measure E4 orf1 gene copies. Fold increase of viral genome copies is expressed in relation to genome copies detected after 2 hours (set to 1). (B) HuH7.5 and BON cells were transduced with viruses at various MOIs (0.001-1). Cell viability was examined 6 days after transduction and expressed in relation to the viability of untransduced cells.

Despite strong reduction of E1A expression, adenovirus may still have the capacity to replicate [17]. We therefore evaluated replication of the miR122-targeted oncolytic viruses by quantitative real-time (QRT) PCR analysis, measuring genomic viral DNA (E4 orf1) copies at different time points after transduction. Replication index was defined as the increase of copy number of progeny viral DNA compared to the copy number obtained 2 hours after transduction (set to 1). Replication of Ad[CgA-E1A-miR122] and Ad[CgA-E1A-miR122×2] was completely attenuated in HuH7.5 without production of progeny viral DNA, in contrast to Ad[CgA-E1A] and Ad5 wt, which have replication indexes of 330 and 7200, respectively, after 3 days (Figure 4A). As expected, miR122-mediated inhibition of replication was not observed for any of the viruses in HepG2 (Figure 4A). Importantly, in carcinoid cells and in BON, the CgA promoter-driven oncolytic viruses, either with or without miR122 target sequences, replicate equally well and follow the pattern of Ad5 wt replication (Figure 4A).

We next verified that Ad[CgA-E1A-miR122] and Ad[CgA-E1A-miR122×2] lyse BON cells to a similar degree as Ad[CgA-E1A]. We found that all cells were killed when transduced at MOI 0.1 (Figure 4B). Ad[CgA-E1A] had weak activity and could kill HuH7.5 cells to a certain degree (cell viability of 65%) when transduced at MOI 1, while Ad[CgA-E1A-miR122] and Ad[CgA-E1A-miR122×2] had no effects (Figure 4B).

### miR122-Detargeting of Adenovirus Leads to Reduced Liver Toxicity

**Figure 5:**
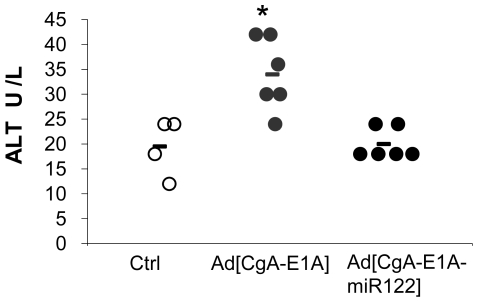
Lack of hepatotoxicity in mice injected with Ad[CgA-E1A-miR122]. Balb/c mice received three intravenous injections with 5×10^10^vp of Ad[CgA-E1A] or Ad[CgA-E1A-miR122] on days 1, 4 and 7. Blood was collected on day 9 and plasma was obtained by incubation blood with heparin (20 U per ml of blood) and used for the measurement of ALT levels (*p = 0,0011).

The Ad[CgA-E1A] and Ad[CgA-E1A-miR122] viruses were next evaluated for liver activity *in vivo*. Viruses (5×10^10^ vp) were injected intravenously (tail vein) on day 1, 4 and 7 and blood was drawn and analyzed on day 9. We found that mice injected with Ad[CgA-E1A] had significantly elevated serum levels of ALT, which is indicative of liver toxicity, while no mice injected with Ad[CgA-E1A-miR122] had elevated ALT levels (Figure 5).

### Combination of Transcriptional (Promoter) and Post-Transcriptional (miRT) Regulation Yields the Most Specific Viruses with the Lowest Activity in Hepatic Cells

**Figure 6:**
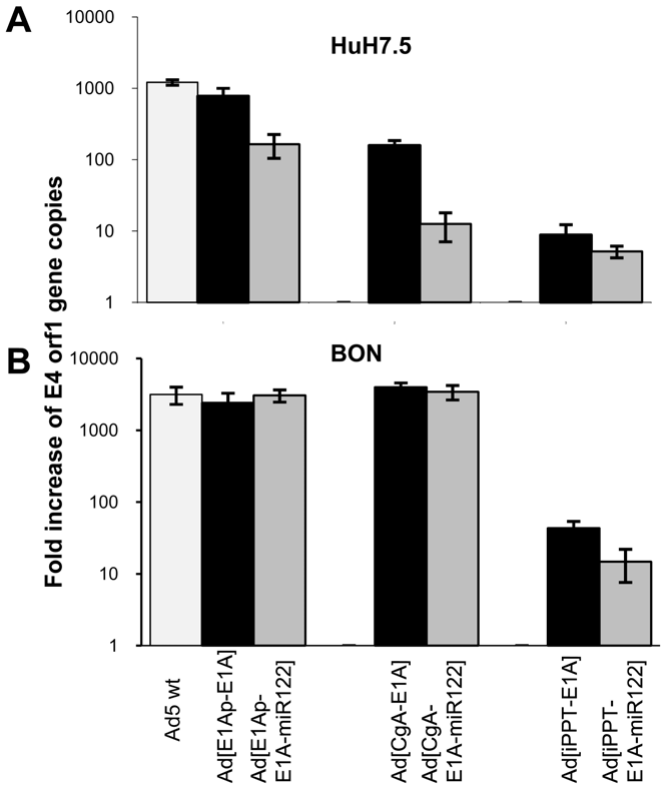
Greater replication arrest by double-targeted than single-targeted adenovirus in hepatic cells. HuH7.5 (A) and BON (B) were transduced at MOI 1. Viral DNA was isolated 2 hours and 2 days after transduction. QRT-PCR was performed to measure E4 orf1 copies. Fold increase of viral genome copies after 2 days is expressed in relation to genome copies detected after 2 hours (set to 1). Data are presented as the mean of triplicates ± std dev.

We finally wanted to extensively assess the benefit of having both promoter and miRT-controlled E1A expression for reduced activity in hepatic cells. We therefore compared the silencing effect of Ad[CgA-E1A-miR122] with two other miR122-detargeted adenoviruses, one where E1A is controlled by the wild-type E1A promoter Ad[E1Ap-E1A-miR122] and one where it is controlled by the prostate cell-specific iPPT promoter, Ad[iPPT-E1A-miR122]. To evaluate the effect of each modification replication efficacies and lytic abilities were also compared with Ad[E1Ap-E1A], Ad[CgA-E1A] and Ad[iPPT-E1A] as well as wild-type Ad5. MiR122-positive hepatic cells (HuH7.5) and miR122-negative carcinoid cells (BON) were transduced at MOI 1 and the gene copy numbers of E4 orf1 were measured by QRT-PCR after two days. The copy numbers were expressed in relation to the copy numbers obtained 2 hours after transduction for the same virus (set to 1), as a measurement of virus replication. The wild-type Ad5 virus replicated best in both cell lines, while the E1B-deleted Ad[E1Ap-E1A] (otherwise wild-type Ad5) virus yielded approximately 90% E4 orf1 gene copy numbers compared to Ad5 wild type both in HuH7.5 and BON, showing that E1B deletion had only minor effects on replication in these cell lines (Figure 6A and 6B). The single targeted Ad[E1Ap-E1A-miR122] and Ad[CgA-E1A] viruses yielded approximately 7-fold and 5-fold less genome copies than Ad5 wt and Ad[E1Ap-E1A], respectively in HuH7.5 (Figure 6A). On the other hand, transduction with the double-targeted Ad[CgA-E1A-miR122] virus yielded 100-fold and 70-fold less genome copies than Ad5 wt and Ad[E1Ap-E1A], respectively, indicating an additive effect of combining promoter targeting with miRT detargeting. In BON cells Ad[E1Ap-E1A], Ad[E1Ap-E1A-miR122], Ad[CgA-E1A] and Ad[CgA-E1A-miR122] yielded similar levels of genome copies (Figure 6B), showing that introduction of miR122 target sequence in the viral genomes does not affect virus replication in miR122 negative cells. Transduction of HuH7.5 and BON with the prostate cell-specific Ad[iPPT-E1A] and Ad[iPPT-E1A-miR122] viruses did not result in significant numbers of genome copies after two days, indicating strongly abolished replication capacity of these viruses in hepatic cells (Figure 6A and 6B). This shows that a tissue-specific promoter alone, if highly specific, can almost completely attenuate virus replication in non-permissive cells.

**Figure 7:**
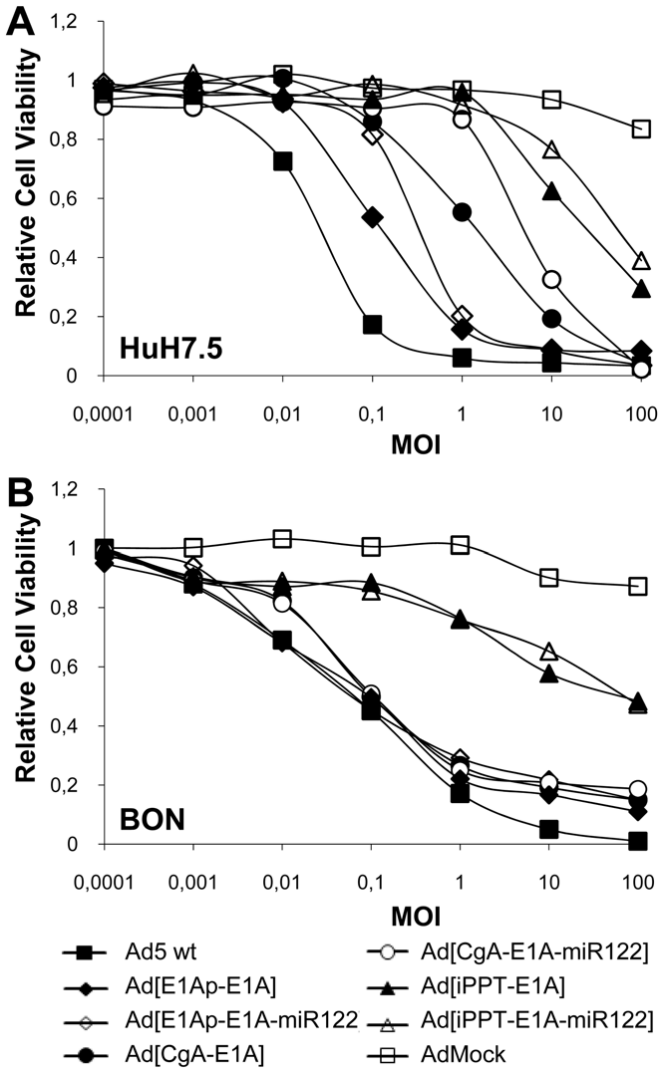
Stronger reduction of cytolytic ability in hepatic cells by double-targeted than single-targeted adenovirus. HuH7.5 (A) and BON (B) were transduced at various MOIs (0.0001-100). Cell viability was examined 6 (HuH7.5) and 4 (BON) days after transduction. Cell viability values were expressed in relation to the viability of untransduced cells. Average enzymatic activities from triplicate samples are shown.

Next, we wanted to examine whether suppressed replication correlates with reduced cell-killing efficacy. An MTS cell viability assay was carried out using HuH7.5 and BON cells transduced with the same set of viruses at MOIs ranging from 0.0001-100. As expected, the Ad5 wt virus was the most efficient in killing HuH7.5 and BON cells, while AdMock did not show lytic ability even at MOI 100 (Figure 7A and 7B). Transduction of HuH7.5 with Ad[CgA-E1A-miR122] at MOI 1 did not reduce cell viability, while Ad5 wt, Ad[E1Ap-E1A], Ad[E1Ap-E1AmiR122] and Ad[CgA-E1A] transduction results in killing of 95%, 85%, 80% and 45% of the cells, respectively (Figure 7A). Ad[CgA-E1A-miR122] and Ad[iPPT-E1A-miR122] start to yield reduced cell viability at MOI 10 and 100, respectively, indicating that at those MOIs the viral E1A mRNA molecules may outnumber cellular miR122 molecules in HuH7.5. Transduction of BON cells at an MOI of 1 results in at least 75% cell killing, for all oncolytic viruses except Ad[iPPT-E1A] and Ad[iPPT-E1A-miR122] (Figure 7B). Our data show that while introduction of a miR122 target sequence in the 3′UTR of the E1A gene leads to reduced killing of hepatic cells, it does not affect the desired killing of miR122-negative target cells.

## Discussion

MicroRNA targeting (miRT) has been used to control transgene expression from viral vectors and viral genes important for replication of oncolytic viruses in tissue-specific manners [6], [8], [9], [10], [18]. An extensive discussion on how to design miRNA target sequences for viral vectors can be found as supporting information Text S1 to this manuscript (miRNA target design). If miRT sequences are placed in a small single-stranded positive-sense (+) RNA genome such as picornaviruses, they become part of the single viral mRNA [8]. miRNA-mediated silencing of adenovirus replication is somewhat more complex due to the large number of transcripts. However, in order to control adenovirus replication by miRNAs, the E1A gene is a good candidate, since it encodes proteins that trigger viral replication and modulate cellular metabolism to make the cell more susceptible to replication. Low levels of E1A protein can be sufficient in supporting adenoviral replication [17]. It may therefore be attractive to introduce miRT sequences in other adenoviral genes, such as E2 or E4, in addition to E1A, in order to further restrict adenoviral replication in the liver and other potential off-target cell types, such as Kupffer cells, monocytes and other hematopoetic cells. Targeting more than one adenoviral gene essential for replication and employing various tissue-specific miRNAs may also protect from loss of activity due to intrinsic miRNA silencing through mutation or deletion of miRT sequences.

In a previous paper, we used the CgA promoter to control E1A expression with the intention to develop an Ad5-based oncolytic adenovirus, Ad[CgA-E1A], for carcinoid therapy [11]. Since midgut carcinoids metastasize to the liver, intrahepatic or intratumoral virus injections would be the most likely route of delivery for treatment of liver metastases. It is therefore crucial that normal liver cells do not support virus replication. We found that CgA promoter-controlled E1A expression results in strong attenuation of E1A protein expression in hepatocytes but that it does not completely abolish expression [11]. In order to further restrict adenoviral replication in hepatocytes we set out to regulate E1A gene expression by the liver-specific miR122. Combined regulation of the E1A gene by the CgA promoter and miR122 target sequences, acts together and can efficiently repress E1A protein expression in cells expressing miR122. It leads to abolished adenoviral replication and reduced cytolytic activity. Moreover, our miR122-targeted oncolytic adenoviruses fully retain replication capacity and killing ability in neuroendocrine tumor cells. There was no difference between the two miRT-modified viruses in terms of replication in hepatocytes, indicating that six copies of miR122 target sequences are sufficient to completely attenuate genes controlled by the CgA promoter. In a recent paper Ylosmaki et al, incorporated miR122 target sequences in the 3′UTR of E1A of an Ad5 virus, which retained the wild-type E1A promoter [10]. They observed decreased E1A protein levels in the hepatoma cell line HuH7 when the cells were transduced at a very low MOI of 0.05. However, this was not sufficient to suppress replication. They managed to improve miR122-responsivness of their recombinant adenovirus by modifying the 5′UTR of the E1A gene. However, it resulted in uniform and unspecific reduction of E1A protein levels in all tested cells. Cawood et al also kept the wild-type E1A promoter and used four miR122 target sequences in the 3′UTR of E1A to reduce Ad5 activity in hepatic cells [9]. They did not report whether insertion of miR122 target sequences was enough to attenuate virus replication in HuH7. Our data demonstrate partial attenuation of replication for Ad[E1Ap-E1A-miR122] (wild-type Ad5 E1A promoter) in HuH7.5, especially at MOI 10. However, complete replication arrest can be obtained as demonstrated by our double-targeted Ad[CgA-E1A-miR122] virus in HuH7.5 and also in normal human hepatocytes. Cawood et al reported reduced liver toxicity in mice after one intravenous injection of 5×10^10^vp for their miR122-detargeted virus compared to wild-type Ad5 [9]. We had to inject 5×10^10^vp of Ad[CgA-E1A] at three occasions before we started to see elevated ALT levels and with the Ad[CgA-E1A-miR122] virus we did not see elevated ALT levels even after three injections.

Furthermore, we made side-by-side comparisons of three miR122-detargeted viruses; one with the wild-type E1A promoter, one with the neuroendocrine cell-selective CgA promoter and one with the prostate cell-specific iPPT promoter. We found that a combination of transcriptional (promoter) and post-transcriptional (miRT) targeting is clearly advantageous in terms of attenuation of virus replication in hepatic cells. Our data suggests that in order to reduce virus replication in hepatic cells, miR122-detargeting alone is effective but not efficient enough to completely attenuate virus replication. It is better to combine it with a promoter that has wild type-like activity in tumor cells but reduced activity in hepatic cells. The combination of transcriptional and post-transcriptional regulation to control virus replication yields a possibility to use higher doses of adenoviruses for more efficient and specific tumor treatment without inducing dose-related liver toxicity. In conclusion, the double-targeted Ad[CgA-E1A-miR122] virus has potential for the treatment of neuroendocrine tumors that metastasize to the liver.

## Materials and Methods

### Ethics Statement

Carcinoid specimens were obtained from patients who underwent liver surgery to remove midgut carcinoid metastases, while wedge biopsies of normal liver were taken from patients who underwent liver surgery for different reasons than carcinoid tumor. A written consent statement was obtained from each patient. All experimental procedures were approved by the Regional Ethical Committee at the Uppsala University Hospital (Regionala etikprövningsnämnden i Uppsala), with reference numbers 2004:M-223 (Hepatocytes) and 2009/152 (Carcinoids). The Uppsala Animal Ethical Committee (Uppsala Djurförsöksetiska nämnd, Ref. no. C52/7) has approved all experimental procedures.

### Cell Lines

The human endocrine pancreatic tumor cell line BON (a kind gift from Prof J.C. Thompson, Galveston, TX) was cultured in DMEM with Glutamax-I and F12 Nutrient Mixture (Kaighn′s modification) at a ratio of 1∶1, supplemented with 10% fetal bovine serum (FBS), 1 mM sodium puruvate and 1% penicillin/streptomycin (PEST). The human neuroblastoma cell lines SH-SY-5Y and SK-N-BE(2) (Dr F. Hedborg, Uppsala University, Uppsala, Sweden) were cultured in MEM with Earl′s salt and L-glutamine, supplemented with 10% FBS, 1 mM sodium puruvate, 1% PEST. The neuroblastoma cell line Kelly was cultured in RPMI-1640 medium, supplemented with 10% FBS, 1 mM sodium puruvate, 1% PEST. The human hepatoma cell lines HuH7.5 (Apath, L.L.C., St.Louis, MO) and HepG2 (Prof N. Maitland, University of York, York, UK) were cultured in DMEM with Glutamax-I, supplemented with 10% FBS, 1 mM sodium puruvate, 3.2 mM L-glutamine, 1% Non-Essential Amino Acids, 1% PEST. The 911 (Crucell, Leiden, The Netherlands) and lung carcinoma A549 (ATCC, Manassas, VA) cell lines, used for virus production, were cultured in DMEM supplemented with 10% FBS, 1 mM sodium pyruvate, 1% PEST. All cell culture reagents were from Invitrogen (Carlsbad, CA).

### Isolation and Culture of Primary Cells

Isolation and culture conditions of normal hepatocytes have been described earlier [11]. Carcinoid tumor specimens were placed in ice-cold rinse buffer [0.1% BSA, 2.5 mM Hepes, DNaseI (0.05 mg/ml), NaCl (800 mg/L), KCl (35 mg/L), MgSO_4_x7H_2_O (16 mg/L), CaCl_2_xH_2_O (18 mg/L)]. Cold ischemic time was 30–60 minutes. Tumor samples were transferred to a sterile Petri-dish and cut into small pieces with a scalpel and placed in digestion buffer [Ham's F-10, Collagenase (2 mg/ml), DNAse I (0.05 mg/ml), 1.5% BSA, 1.25 mM CaCl_2_] for 40 min at 37°C, while shaking. The digested tumor specimens were filtered through a sterile metal grid (mesh size 440 µm) with rinse buffer, collected and centrifuged (300×g, 10 min, RT). The supernatant was removed and cells were resuspended in 0.5 ml EGTA buffer [NaCl (8.3 g/L), KCl (0.5 g/L), 25 mM Hepes, EGTA (0.38 g/L)] and mixed with 5 ml rinse buffer. Five ml 60% Percoll solution (GE Healthcare Life Sciences, Uppsala, Sweden) was added to the bottom of the tube, followed by centrifugation (300×g, 10 min, RT). The middle phase was transferred into a new tube followed by addition of 5 ml rinse buffer. Five ml 25% Percoll solution was added to the bottom of the tube and the cells were centrifuged (300×g, 10 min, RT). The supernatant was removed and cells were resuspended in HITES medium [RPMI-1640, supplemented with 1% ITS-G (Invitrogen), estradiol (0.24 mg/ml), hydrocortisol (0.36 mg/ml), 5% FBS (Invitrogen), 1% L-glutamine (Invitrogen), 1% PEST (Invitrogen)]. Cells were plated in T-75 cell-bind flasks (Corning, Lowell, MA). The carcinoid cells were rigidly flushed and collected the following day, leaving a small number of fibroblast adherent in the flasks. The carcinoids cells were trypsinized and plated in fresh T-75 cell-bind flasks overnight. Cells were used for experiments the day after, i.e., 48 hours after surgery. All chemicals were from Sigma-Aldrich (St. Louis, MO), except when stated differently.

### Recombinant Adenoviruses

The recombinant adenoviruses in this study are E1B-deleted and based on human serotype 5 (Ad5) and constructed using AdEasy technology. The Ad5 backbone vector pAdEasy(E3) and the transfer vector pShuttle-i/CgA-E1A, which were used for construction of Ad[CgA-E1A] have been described earlier [11], [19] and so has the constructions of AdMock and Ad[CMV-Luc] [20] and the prostate-specific Ad[iPPT-E1A] [19]. The Ad5 E1A gene was PCR amplified from wild-type Ad5 DNA using the following primers: E1A.for 5′-AAG CTT CTC TTG AGT GCC AGC GAG TA-3′ (HindIII site underlined); E1A.rev: 5′-GTC GAC CCA TTT AAC ACG CCA TGC AAG T-3′ (SalI site underlined), followed by TOPO TA-cloning into pCR2.1 (Invitrogen), sequencing and HindIII/SalI subcloning into pShuttle(i/CgA-E1A) where it replaced the old SalI/SalI-cloned E1A sequence. A synthetic dsDNA sequence containing six tandem repeats, complementary to the mature hsa-miR-122 target sequences (http://microrna.sanger.ac.uk/) with six nucleotide spacers between each repeat, and flanked by XbaI sites and an upstream HpaI site and a downstream SmaI site was purchased in a pGA4 plasmid (GeneArt, Regensburg, Germany). The miR122 target sequence was excised from the pGA4 plasmid with HpaI and SmaI and blunt-end cloned into the unique HpaI site in the 3′UTR region of E1A of pShuttle(i/CgA-E1A), located approximately 30 bp downstream of the E1A stop codon and 40 bp upstream of the E1A polyadenylation signal. We sequenced two clones with the insert in the correct orientation, one with six miR122 target sequences: pShuttle(i/CgA-E1A-miR122) and one with 12 tandem repeats of the miR122 target sequence: pShuttle(i/CgA-E1A-miR122×2). Furthermore, the miR122 target sequence was excised from the pGA4 plasmid and cloned into the unique XbaI site directly downstream of the luciferase stop codon in pShuttle vectors. The E1A promoter was amplified from wild-type Ad5 DNA (ATCC) using the following primers: E1Aprom.for 5′-GGT ACC GGG CCG CGG GGA CTT TG-3′ (KpnI site underlined); E1Aprom.rev: 5′-AAG CTT TGG CCT CTT GAG GAA CTC AC-3′ (HindIII site underlined) followed by TOPO TA-cloning into pCR2.1 (Invitrogen) and sequencing. pShuttle(E1Ap-E1A) and pShuttle(E1Ap-E1A-miR122) were created by replacing the iCgA promoter with the E1A promoter using KpnI and HindIII. The pShuttle(i/PPT-Luc) plasmid [19] was digested with XbaI (unique site directly downstream of the luciferase stop codon), made blunt end by Klenow fragment, followed by insertion of the HpaI/SmaI-excised miR122 target sequences (six copies) to construct pShuttle(i/PPT-Luc-miR122). The PPT promoter was replaced with the CgA promoter both in pShuttle(i/PPT-Luc) and pShuttle(i/PPT-Luc-miR122) using NotI and HindIII to construct pShuttle(i/CgA-Luc) and pShuttle(i/CgA-Luc-miR122). Plasmids with full length E1B-deleted adenovirus genomes: Ad[CgA-Luc], Ad[CgA-Luc-miR122], Ad[CgA-E1A], Ad[CgA-E1A-miR122], Ad[CgA-E1A-miR122x2], Ad[E1Ap-E1A], Ad[E1Ap-E1A-miR122] and Ad[iPPT-E1A-miR122] were obtained through homologous recombination in BJ5183 between pAdEasy(E3) [19] and described pShuttle plasmids. Wild-type Ad5 was purchased from ATCC and amplified in A549 cells while recombinant viruses were produced in 911 cells. Viruses were purified by CsCl banding and dialyzed against a buffer containing 10 mmol/L Tris-HCL (pH 8.0), 2 mmol/L MgCl_2_ and 4% sucrose. Virus titers were determined by a fluorescence-forming unit (FFU) assay and viral particle (vp) concentrations were determined by spectrophotometer using standard protocols. The recombinant viruses and wild-type Ad5 (Ad5wt) were stored in aliquots in −80°C.

### Analysis of Endogenous miR122 Expression

Total RNA was extracted from cell lines and freshly isolated carcinoid cells by using Trizol (Invitrogen). Total RNA from normal human liver and Balb/c mouse liver were obtained from 15–20 cryosections (20 µm), which were added to 600 µl of the RLT lysis buffer from the RNeasy Mini Kit (Qiagen, Hilden, Germany). Reverse transcriptase reactions were performed using 1 µg of total RNA of each sample and the QuantiMir RT Kit (System Biosciences, Mountain View, CA), according to the manufacturer's instructions. cDNA (diluted 1∶100) was used for QRT-PCR together with the miR122.For primer 5′-TGG AGT GTG ACA ATG GTG TTT G-3′ and the 3′ Universal Primer provided by the QuantiMir RT Kit. Human and mouse U6 snRNA were amplified with primers provided from the QuantiMir RT Kit. The PCR products were measured by iCycler IQ real-time detection system using iQ SYBRGreen supermix (Bio-Rad, Hercules, CA). Data were evaluated using the 2^−ΔΔCT^ method [21], [22]. The miR122 expression levels were normalized against the U6 snRNA transcript levels.

### Luciferase Assay

Cells were transduced in suspension with Ad[CgA-Luc] and Ad[CgA-Luc-miR122] at a multiplicity of infection (MOI) of 10 FFU/cell. Two hours after transduction cells were plated in 12-well plates. Some cells were subsequently transfected with 10 nM of chemically synthesized siRNA that acts as hsa-mir-122 (miRIDIAN Mimic, Thermo Scientific Dharmacon, Chicago, IL) or non-related siRNA (miRIDIAN Negative Control) using INTERFERin (Polyplus Transfection, New York, NY) according to the manufacturer's protocol. Total lysates were obtained after 48 hours and luciferase assays were performed as described earlier [19]. Luciferase activity was measured and calculated as raw light unit (RLU) per mg of protein and expressed in relation to Ad[CgA-Luc], which for each cell type was set to 100%.

### Animal Studies

Female Balb/c mice, 6–8 weeks of age (B&K Universal, Sollentuna, Sweden), were injected intravenously (tail vein) with 5×10^10^vp of Ad[CMV-Luc], Ad[CgA-Luc] or Ad[CgA-Luc-miR122] in 200 μl of buffer (10 mM Tris-HCL (pH 8.0), 2 mM MgCl_2_ and 4% sucrose). Luciferase activity was measured 6, 24 and 48 hours after injection as described before [11]. Counter regions of interest (ROIs) were created and signals were quantified as photons per second per square centimeter per steradian. For liver toxicology studies, female Balb/c mice were injected in the tail vein on days 1, 4 and 7 with 5×10^10^vp of Ad[CgA-E1A] or Ad[CgA-E1A-miR122]. On day 9, blood was collected by cardiac puncture. Plasma was obtained by incubation of 600 µl of blood with 12 U of heparin for 15 min at RT followed by centrifugation for 20 min at 4°C, 3000×g. Alanine aminotransferase (ALT) levels were measured at the Clinical Chemistry Lab (University Animal Hospital, Uppsala, Sweden).

### Western Blot

Cells cultured on six-well plates were transduced at 60–80% confluency with viruses at MOIs of 1 or 10 FFU/cell. Cells were harvested 48 hours later and total protein extracts were prepared as described earlier [20]. E1A protein expression was determined as described earlier [11] using 50 µg of total lysates. The membranes where then stripped, washed and incubated with α-Prage [23], which is rabbit serum against adenoviral capsid proteins (a kind gift from Dr G. Akusjärvi, Uppsala University, Uppsala, Sweden).

### Replication Assay

Cells were transduced in suspension with viruses at 1 or 10 FFU/cell. Two hours after transduction cells were washed and plated in 24-well plates (2.5×10^5^ cells per well). Cell were harvested directly (day 0) and 1, 2, 3 days after transduction, followed by viral DNA isolation using the High Pure Viral Nucleic Acid Kit (Roche Applied Sciences, Mannheim, Germany) according to the manufacturer's protocol. Viral replication was detected by QRT-PCR with primers for the adenoviral E4 orf1 transcript: E4.For 5′-CAT CAG GTT GAT TCA CAT CGG-3′ and E4.Rev 5′-AAG CGC TGT ATG TTG TTC TG-3′. Gene-specific PCR products were measured by iCycler IQ real-time detection system (Bio-Rad, Hercules, CA) using iQ SYBR Green supermix (Bio-Rad). The data were evaluated using the 2^−ΔΔCT^ method [21], [22] using the DNA level of E4 gene from day 0 from each sample for normalization.

### Cell Viability Assay

Cells were transduced in suspension with viruses at MOIs of 0.0001-100, FFU/cell. Two hours after transduction, the cells were plated in 96-well plates (1×10^4^ cells per well). Cell viability was analyzed 4 or 6 or after transduction using the MTS cell titer 96 aqueous one solution cell proliferation assay (Promega, Madison, WI) according to the manufacturer's instructions.

### Statistical Analysis

Significance was evaluated using one-way ANOVA (GraphPad Software, San Diego, CA) and denoted in the graphs as * p<0.01 and *** p< 0.0001.

## Supporting Information

Text S1 miRNA target design
(0.04 MB DOC)

Figure S1 Analysis of endogenous miR122 expression. cDNAs were synthesized using 1 µg of total RNA from normal human liver, Balb/c mice liver and a panel of cell lines using the QuantiMir kit. QRT-PCR was performed to detect miR122 transcripts and Ct values were calculated from triplicate samples, data were evaluated using the 2^−ΔΔCT^ method and expressed in relation to the level of snRNA U6.
(0.01 MB PDF)

Figure S2 miR122-specific silencing of luciferase expression. Freshly isolated primary cells and cell lines were transduced with Ad[CgA-Luc] and Ad[CgA-Luc-miR122] at MOI 10 and plated in 12-well plates. Cells were harvested after 48 hours and luciferase activity and protein concentration were determined. Luciferase activity was calculated as RLU/mg. Data are presented as the mean values of triplicates ± SD.
(0.01 MB PDF)

Figure S3 Specific silencing of luciferase transgene by mimic siRNA. Cells were transduced with Ad[CgA-Luc] and Ad[CgA-Luc-miR122] at MOI 10 and plated in 12-well plates. In addition, cells were co-transfected with 10 nM of mimic siRNA (acting as hsa-miR-122) or negative control siRNA (non-related). Cells were harvested after 48 hours and luciferase assay and protein concentration measurements were performed. Luciferase activity was calculated as RLU/mg and expressed in relation to Ad[CgA-Luc] which was set to 100%, for each cell type. Transduction with Ad[CgA-Luc] and co-transfection with negative or mimic siRNA, yielded no difference in luciferase activity compared to Ad[CgA-Luc] alone for any of the cells (data not shown).
(0.06 MB PDF)
