# Supplementary material for: Double-Detargeted Oncolytic Adenovirus Shows Replication Arrest in Liver Cells and Retains Neuroendocrine Cell Killing Ability
Source: PLoS One. 2010 Jan 27;5(1):e8916. doi: 10.1371/journal.pone.0008916 (PMC2811733; doi:10.1371/journal.pone.0008916)
Supplement: Text S1 — miRNA target design (0.04 MB DOC) [file pone.0008916.s001.doc]

A new strategy to control viral replication is based on gene silencing has emerged, where viral mRNA levels are altered by tissue-specific cellular miRNAs. This strategy is applicable both for DNA and RNA viruses. Furthermore, since miRT sequences are short they can easily fit into compact viral genomes. However, several features need to be taken into consideration for miRT sequence design. First, only one of the strands of the miRNA duplex, usually the one which 5’end is less tightly paired, is incorporated into the RNA-induced silencing complex (RISC) and binds to target mRNA. The level of homology between miRNA and mRNA can differ and it has been proposed that perfect base pairing in the seed region (usually position 2-8 from the 5’end of the miRNA strand) triggers mRNA cleavage, while partial complementarity between miRNA and mRNA causes translational blockage (1). One fully complementary site may be sufficient for miRNA-induced cleavage, while translational blockage requires multiple imperfect sites recognized by the same or several different miRNAs (1).

The optimal position and optimal number of repeats of miRNA target sequence (miRT) that should be included in viral vectors are currently not known. We used either 6 or 12 repeats with equally good results. We separated the miRT sequences with 6 bp spacers, which gives approximately 18 bp distances between seed pairing region of each repeat. It has been reported that if miRT sequences are too close to each other, gene silencing may be less effective. The strongest repression of target mRNA was observed with 8-40 bp spacing between multiple miRNA seed sites (2). Most cellular mRNAs with known miRT sequences have the miRT sequences in the 3’UTR, although miRT sequences in 5’UTRs can also be effective in gene silencing (3). Moreover, chemically synthesized siRNAs can successfully suppress gene expression, targeting any region of the mRNA (4). It has been hypothesized that the exact location of miRT sequences is not as important as the secondary structures of the mRNA. Regions with a great deal of secondary structure are not well recognized by miRNAs. Furthermore, it has been shown that if miRT sites are located too close to the stop codon (seed region within 15 bp), it may result in poor gene silencing efficacy (2). Our miRT-containing adenoviral constructs fulfills the described determinants.

**REFERENCES**

1. Pillai RS, Bhattacharyya SN, Filipowicz W. Repression of protein synthesis by miRNAs: how many mechanisms? Trends Cell Biol 2007;17:118-26.

2. Grimson A, Farh KK, Johnston WK, et al. MicroRNA targeting specificity in mammals: determinants beyond seed pairing. Mol Cell 2007;27:91-105.

3. Barnes D, Kunitomi M, Vignuzzi M, Saksela K, Andino R. Harnessing endogenous miRNAs to control virus tissue tropism as a strategy for developing attenuated virus vaccines. Cell Host Microbe 2008;4:239-48.

4. Manoharan M. RNA interference and chemically modified small interfering RNAs. Curr Opin Chem Biol 2004;8:570-9.
