# Supplementary figures and images for: Double-Detargeted Oncolytic Adenovirus Shows Replication Arrest in Liver Cells and Retains Neuroendocrine Cell Killing Ability
Source: PLoS One. 2010 Jan 27;5(1):e8916. doi: 10.1371/journal.pone.0008916 (PMC2811733; doi:10.1371/journal.pone.0008916)

Supporting Figure S1

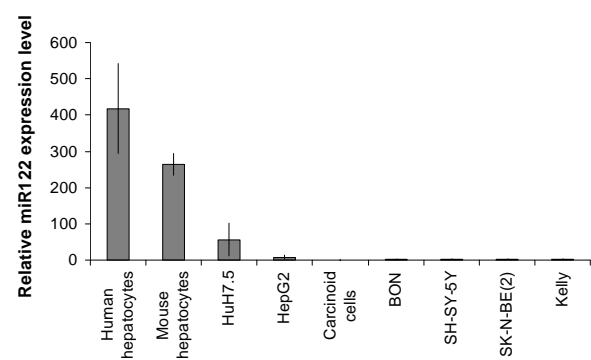

Supplement: Figure S1 — Analysis of endogenous miR122 expression. cDNAs were synthesized using 1 µg of total RNA from normal human liver, Balb/c mice liver and a panel of cell lines using the QuantiMir kit. QRT-PCR was performed to detect miR122 transcripts and Ct values were calculated from triplicate samples, data were evaluated using the 2−ΔΔCT method and expressed in relation to the level of snRNA U6. (0.01 MB PDF) [file pone.0008916.s002.pdf]

Supporting Figure S3

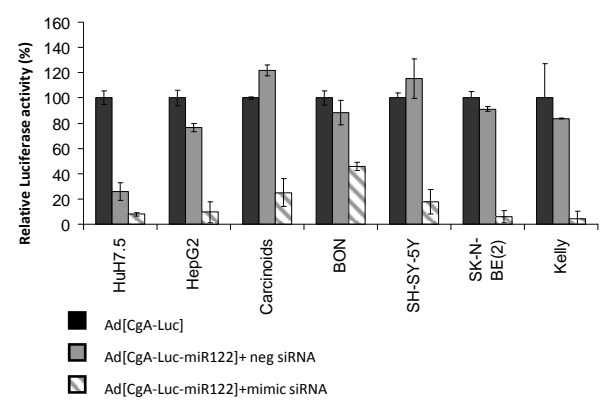

Supplement: Figure S3 — Specific silencing of luciferase transgene by mimic siRNA. Cells were transduced with Ad[CgA-Luc] and Ad[CgA-Luc-miR122] at MOI 10 and plated in 12-well plates. In addition, cells were co-transfected with 10 nM of mimic siRNA (acting as hsa-miR-122) or negative control siRNA (non-related). Cells were harvested after 48 hours and luciferase assay and protein concentration measurements were performed. Luciferase activity was calculated as RLU/mg and expressed in relation to Ad[CgA-Luc] which was set to 100%, for each cell type. Transduction with Ad[CgA-Luc] and co-transfection with negative or mimic siRNA, yielded no difference in luciferase activity compared to Ad[CgA-Luc] alone for any of the cells (data not shown). (0.06 MB PDF) [file pone.0008916.s004.pdf]
