# Supplementary material for: Double-Detargeted Oncolytic Adenovirus Shows Replication Arrest in Liver Cells and Retains Neuroendocrine Cell Killing Ability
Source: PLoS One. 2010 Jan 27;5(1):e8916. doi: 10.1371/journal.pone.0008916 (PMC2811733; doi:10.1371/journal.pone.0008916)
Supplement: Figure S2 — miR122-specific silencing of luciferase expression. Freshly isolated primary cells and cell lines were transduced with Ad[CgA-Luc] and Ad[CgA-Luc-miR122] at MOI 10 and plated in 12-well plates. Cells were harvested after 48 hours and luciferase activity and protein concentration were determined. Luciferase activity was calculated as RLU/mg. Data are presented as the mean values of triplicates ± SD. (0.01 MB PDF) [file pone.0008916.s003.pdf]

Supporting Figure S2

Cell lines	Ad[CgA-Luc]		Ad[CgA-Luc-miR122]	
	Luciferase activity (RLU/mg)	±SD	Luciferase activity (RLU/mg)	±SD
**Hepatocytes**	1208	90	50	28
**Carcinoids**	1791	15	2661	208
**HUH7.5**	261433	14214	68319	2346
**HepG2**	3318935	210922	3868343	235106
**BON**	350385	19677	426943	115189
**SH-SY-5Y**	353872	14613	380207	52749
**SK-N-BE(2)**	168839	8787	203452	7486
**Kelly**	25999	7064	20664	2530
